# Polymorphisms in matrix metalloproteinases 2, 3, and 8 increase recurrence and mortality risk by regulating enzyme activity in gastric adenocarcinoma

**DOI:** 10.18632/oncotarget.22516

**Published:** 2017-11-20

**Authors:** Youdong Lin, Jinsheng Liu, Long Jin, Yun Jiang

**Affiliations:** ^1^ Fujian Shengli Clinical Medical College of Fujian Medical University and Department of Clinical Laboratory Medicine, Fujian Provincial Hospital, Fuzhou, Fujian 350001, China; ^2^ Department of Gastrointestinal Surgery, Fujian Provincial Hospital, Fuzhou, Fujian 350001, China; ^3^ Department of Pathology, Fujian Provincial Hospital, Fuzhou, Fujian 350001, China; ^4^ Department of VIP Clinic, Fujian Provincial Hospital, Fuzhou, Fujian 350001, China

**Keywords:** matrix metalloproteinases, polymorphisms, enzyme activity, gastric adenocarcinoma, clinical outcomes

## Abstract

The association of polymorphisms in matrix metalloproteinases (MMPs) with clinical outcomes of gastric adenocarcinoma has not been examined. Ten polymorphisms in MMP1, 2, 3, 7, 8, 9, 12, and 13 were genotyped and investigated, and patients were followed for an average of 58 months. The activities of MMP2, 3, and 8 were measured. Recurrence risk increased in patients with the *MMP2 rs2285053* CC genotype (hazard ratio [HR], 1.85), *MMP3 rs679620* AA genotype (HR, 2.15), and *MMP8 rs1940475* TT genotype (HR, 2.22) on recurrence free survival (RFS). Co-presence of the unfavorable *MMP2 rs2285053* CC and *MMP8 rs1940475* TT genotypes resulted in an additional increased risk of recurrence (RFS: HR, 4.42; 95% confidence interval [CI], 2.15–9.09; p<0.0001) and risk of death (overall survival ( OS) : HR, 6.59; 95% CI, 3.15–13.19; p<0.0001). Theoretical survival tree analysis revealed that recurrence-free survival significantly varied from 15.5 to 87 months among patients with different polymorphisms in MMP2, 3, and 8. The enzymatic activities of MMP2 and MMP3 increased (*MMP2 rs2285053* CC: 888.60 vs. CT: 392.00, p <0.0001; *MMP3 rs679620* AA: 131.10 vs. GG: 107.74, p=0.015), whereas those of MMP8 decreased (*MMP8 rs1940475* TT: 133.78 vs. CC: 147.54, p=0.011) in gastric cancer tissues. These results suggest that polymorphisms in MMP2, 3, and 8 may increase cancer recurrence and patient death by increasing or decreasing enzyme activity in patients with gastric adenocarcinoma.

## INTRODUCTION

Gastric cancer is the third most common cause of cancer-related deaths worldwide. There are approximately 989,600 new gastric cancer cases and 738,000 deaths annually, accounting for 8% of new cancer cases and 10% of total cancer-related deaths, respectively [1]. The majority of gastric cancers are gastric adenocarcinomas (GAs). Surgery remains the primary treatment for this disease, and postoperative chemotherapy is a common clinical practice except in very early-stage cancers [2]. Similar to many other cancers, fluoropyrimidines and platinum compounds are currently the first-line chemotherapy drugs. The outcome of GA significantly varies even in patients with a similar stage and degree of disease, indicating that genetic and epigenetic variations may be important contributors to GA. Single nucleotide polymorphisms (SNPs) are common genetic variations that may directly cause differences in gene expression and protein function, resulting in altered disease pathogenesis and different pharmacokinetic responses to chemotherapy [3–6].

Matrix metalloproteinases (MMPs) are a group of endopeptidases in the metzincin protease superfamily that mainly function to degradevarious extracellular matrix (ECM) proteins such as collagen, laminin, and elastin. They are also actively involved in ECM turnover, wound healing, and tissue homeostasis. The aberrant expression and activation of MMPs occur in several different types of human cancers including GA [7–10], in part due to their ability to regulate non-ECM molecules such as intercellular adhesion molecules, growth factor precursor, cytokines, and cytokine receptors, to increase cancer cell proliferation [11, 12]. Previous studies have shown that SNPs in MMP genes are associated with different clinical outcomes of breast cancer [13]. Moreover, studies about ovarian and non-small cell lung cancers demonstrated that SNPs in MMPs directly increase cell sensitivity and tissue toxicity of platinum compounds and fluoropyrimidines [3, 14]. MMPs are also involved in gastric cancer growth and metastasis. In fact, the expression of MMP2, 7, and 9 is significantly elevated in gastric cancer tissues, and the silencing of MMP3 expression in gastric cancer cells decreased GA invasiveness. In this study, we investigated the association of SNPs in MMP1, 2, 3, 7, 8, 9, 12, and 13 with clinical outcomes in patients with GA.

## RESULTS

### Patient characteristics

General characteristics of the 254 patients with GA are shown in Table 1. Patients were treated with two to five cycles of fluoropyrimidine-based chemotherapy after surgery. At follow-up, 140 (55.1%) recurrence and 129 (50.8%) patient death had occurred. The median recurrence and survival time were 41 months and 61 months, respectively. As expected, tumor stage was the most significant factor related to recurrence and survival (p<0.001). Somewhat surprisingly, no other common clinical variable, including older age (≥65 years old), was significantly associated with recurrence and survival (Table 1). Hardy–Weinberg equilibrium tests were performed (Table 2). Additionally, chi-square test showed that patients with different SNPs had similar demographics and disease characteristics (Supplementary Table 2).

**Table 1:** Patients characteristics

Characteristics	n (N=254)	%	Survival		Recurrence	
			N (Alive/Dead)	P^*^	N (No/Yes)	P^*^
**Age**				0.140		0.318
< 65	193	76	100 / 93		90 / 103	
≥65	61	24	25 /36		24 / 37	
**Gender**				0.587		0.420
Male	183	72.0	92 / 91		85 / 98	
Female	71	28.0	33 / 38		29 / 42	
**Histologic grade**				0.270		0.547
Well differentiated	16	6.3	7 / 9		7 / 9	
Moderately differentiated	158	62.2	84 / 74		75 / 83	
Poorly differentiated	80	31.5	34 / 46		32 / 48	
**Gross type**				0.138		0.383
Superficial	4	1.6	4 / 0		3 / 1	
Apophysis	18	7.1	9 / 9		9 / 9	
Invasion	231	90.9	111 / 120		101 / 130	
Massive type	1	0.4	1 / 0		1 / 0	
**Tumor location**				0.394		0.421
Cardiac	35	13.8	15 / 20		12 / 23	
Gastric fundus	4	1.6	2 / 2		2 / 2	
Gastric body	12	4.7	9 / 3		7 / 5	
Gastric antrum	198	78.0	96 / 102		92 / 106	
Whole stomach	5	2.0	3 / 2		1 / 4	
**Chemotherapy**				0.139		0.392
Fuoropyrimidine only	76	29.9	32/44		31/45	
Fuoropyrimidine+Platinum	178	70.1	93/85		83/95	
**Survival** (Alive / Dead)	125 / 129	49.2 / 50.8				
**Recurrence** (No / Yes)	114 / 140	44.9 / 55.1				
**TNM stage**				**<0.001**		**<0.001**
1	54	21.3	48/6		43/11	
2	73	28.7	33/40		32/41	
3	111	43.7	41/70		37/74	
4	16	6.3	3/13		2/14	

^*^p<0.05 was considered significant and are depicted in bold.

**Table 2:** Ten genotyped gingle-nucleotide polymorphisms of the MMPs gene

Gene and NCBI SNP ID	Chromosome	Location	Base change	Genotyping rate, %	HWE test	Minor allele frequency		
						In current data	CHB^a^	CEU^a^
MMP1 rs1799750	11	102175706	1G →2G	96.5	0.690	0.37	0.451^b^	
MMP2 rs2285053	16	54069878	C→T	100	0.330	0.24	0.173^b^	
MMP2 rs243865	16	54069307	C→T	99.6	0.510	0.11	0.08	0.25
MMP3 rs679620	11	102218830	A →G	100	0.080	0.34	0.32	0.41
MMP7 rs11568818	11	101906871	A→G	100	0.047	0.08	0.09	0.47
MMP8 rs1940475	11	102098458	C→T	98.8	0.540	0.38	0.42	0.49
MMP9 rs17576	20	44073632	G→T	99.2	0.120	0.30	0.37	0.27
MMP9 rs2250889	20	44075813	C→G	99.2	0.800	0.24	0.28	0.05
MMP12 rs2276109	11	102251001	A→G	99.2	0.001	0.048	0.065	0.11
MMP13 rs2252070	11	102331749	A→G	99.2	0.500	0.46	0.49	0.31

A, adenine; C, cytosine; G, guanine; T, thymidine; CHB, Han Chinese in Beijing, China; CEU, the Offspring of the descents from north European and Western European populations In Utah, USA. ^a^ Frequency was determined by the HapMap Project; ^b^ Frequency was determined by the dbSNP irrespective of population.

### Association of SNPs with recurrence risk

We assessed the association between SNPs in *MMP1, 2, 3, 8, 9*, and *13* and recurrence risk in GA patients. The results showed that SNPs in *MMP2* rs2285053, *MMP3 rs679620*, and *MMP8 rs1940475* were associated with increased recurrence risk (Table 3). Because *MMP1, 3, 7, 12*, and *13* genes are co-located on chromosome 11q22.3, the likely contribution of their haplotypes was examined. However, none of the SNP haplotypes was associated with recurrence risk (data not shown). There are three *MMP2 rs2285053* genotypes, namely CT, CC, and TT. Because only 17 patients had the TT genotype, our analysis focused on the difference between the CC and CT genotypes. A higher recurrence risk was observed in patients with the CC genotype (recurrence free survival (RFS): hazard ratio [HR], 1.85; 95% confidence interval [CI], 1.19–2.85; p=0.006). Among patients with the *MMP3 rs679620* AA, AG, or GG genotype, recurrence risk was significantly increased in those with the AA genotype (RFS: HR, 2.15; 95% CI, 1.04–4.45; p=0.040). In patients with the *MMP8 rs1940475* CC, CT, or TT genotype, those with the TT genotype had increased recurrence risk (RFS: HR, 2.22; 95% CI, 1.27–3.87; p=0.005, Table 3).

**Table 3:** MMPs genotypes and recurrence risk

Gene and SNP	Genotype	Recurrence No(n)/Yes (n)	Recurrence rate(%)	RFS		
				HR^#^	95% CI	P^*^
Total		114/140	55.1			
MMP2: rs2285053	CT	75/76	50.3	1 (ref)		
	CC	36/50	58.1	1.85	1.19 to 2.85	**0.006**
	TT	3/14	82.4	2.87	1.42 to 5.83	**0.003**
MMP3: rs679620	GG	52/64	55.17	1 (ref)		
	AG	54/48	47.1	0.72	0.42 to 1.23	0.233
	AA	8/28	77.8	2.15	1.04 to 4.45	**0.040**
MMP8: rs1940475	CC	51/47	48.0	1 (ref)		
	CT	49/65	57.0	1.54	0.97 to 2.45	0.066
	TT	11/28	71.8	2.22	1.27 to 3.87	**0.005**

SNP, single nucleotide polymorphism; MMP, matrix metalloproteases; HR, hazard ratio; ref, reference; RFS, recurrence free survival.

^#^ Adjusted for age, gender, histologic grade, gross type, tumor location, TNM stage and chemotherapy regimens. ^*^p<0.05 was considered significant and were depicted in bold. HR and P values were obtained by multivariate Cox Proportional analysis.

### Cumulative effects of two unfavorable SNPs on recurrence risk

**Figure 1:**
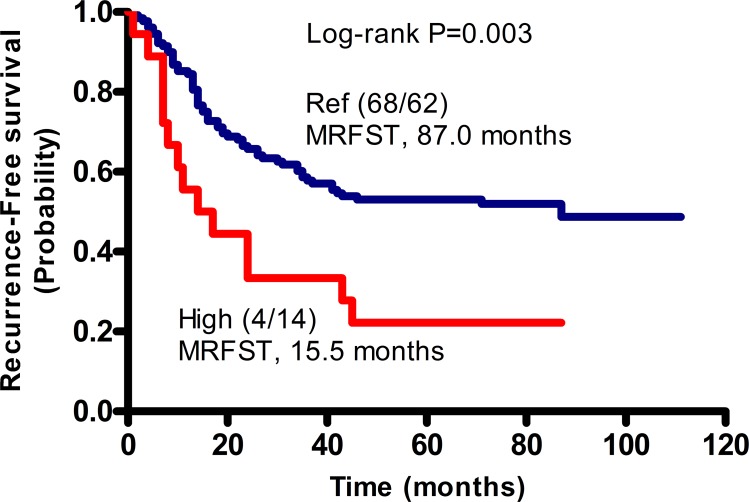
Cumulative effects of unfavorable SNPs on the median recurrence-free survival time (MRFST) in patients with GA Kaplan-Meier analysis was performed. Patients without unfavorable genotype, namely the CT genotype of MMP2 rs2285053 and the CC or CT genotype of MMP8 rs1940475 were arbitrarily set as reference control (Ref). Patients with combined unfavorable genotypes containing CC of MMP2 rs2285053 and TT of MMP8 rs1940475 had a significantly shortened MRFST, and were therefore defined as the high risk group (High). In parentheses, numerator represents the number of patients without recurrence and denominator represents the number of patients with recurrence.

As mentioned above, recurrence risk was relatively high in patients with the *MMP2* rs2285053 CC, *MMP8* rs1940475 TT, and *MMP3* rs679620 AA genotypes. To understand the correlation between these genotypes and recurrence risk, we evaluated patients with the combined genotype of two or three unfavorable SNPs. Because patients with the *MMP2* rs2285053 CT and *MMP8* rs1940475 CC/CT genotypes had a relatively low recurrence rate (47.7%), they were set as the reference group (Table 4). As expected, patients harboring both unfavorable genotypes of *MMP2* rs2285053 CC and *MMP8* rs1940475 TT had a significantly increased recurrence risk and were identified as the high risk group (recurrence rate: 77.8%; RFS: HR, 4.42; 95% CI, 2.15–9.09; p<0.0001). Kaplan–Meier analysis revealed that patients with the *MMP2* rs2285053 CT and *MMP8* rs1940475 CC/CT genotypes had a median-recurrence free survival time (MRFST) of 87 months, whereas patients with the combined *MMP2* rs2285053 CC and *MMP8* rs1940475 TT genotypes had a significantly shorter MRFST of 15.5 months (p=0.003; Figure 1).

**Table 4:** Cumulative effect of multiple SNPs and risk for recurrence

Group	Recurrence No(n)/Yes (n)	Recurrence rate (%)	RFS		
			HR^#^	95% CI	P^*^
Reference^§^	68/62	47.7	1 (ref)		
High risk ^§^	4/14	77.8	4.42	2.15 to 9.09	**<0.0001**

SNP, single nucleotide polymorphism; HR, hazard ratio; ref, reference; RFS, recurrence free survival.

^#^Adjusted for age, gender, histologic grade, gross type, tumor location, TNM stage and chemotherapy regimens. ^*^p<0.05 was considered significant and were depicted in bold. HR and P values were obtained by multivariate Cox Proportional analysis.

^§^Reference: harbing CT genotype of MMP2 rs2285053 and CC/CT genotype of MMP8 rs1940475. ^§^High risk: the co-presence of CC genotype of MMP2 rs2285053 and TT genotype of MMP8 rs1940475.

### Association of SNPs with survival

A total of 129 patients died during the 58-month follow-up period, resulting in an overall mortality of 50.8%. Different SNPs in *MMP2 rs2285053*, *MMP3 rs679620*, *MMP8 rs1940475*, and *MMP13 rs2252070* were associated with different survival times (Table 5). Consistent with the increased recurrence risk, a significant decrease in survival was observed in patients with the *MMP3 rs679620* AA genotype (death rate: 69.4%; overall survival (OS): HR, 3.25; 95% CI, 1.50–7.02; p=0.003). Additionally, *MMP2 rs2285053* CC, *MMP8 rs1940475* TT, and *MMP13 rs2252070* GG genotypes were associated with decreased survival (Table 5).

**Table 5:** MMPs genotypes and survival

Gene and SNP	Genotype	Survival Alive (n)/Death (n)	Death rate (%)	OS		
				HR^#^	95% CI	P^*^
Total		125 / 129	50.8			
MMP2: rs2285053	CT	81/70	46.4	1 (ref)		
	CC	36/50	58.1	2.03	1.29 to 3.20	**0.002**
	TT	8/9	52.9	1.77	0.81 to 3.89	0.152
MMP3: rs679620	GG	57/59	50.9	1 (ref)		
	AG	57/45	44.1	0.83	0.13 to 0.57	0.516
	AA	11/25	69.4	3.25	1.50 to 7.02	**0.003**
MMP8: rs1940475	CC	55/43	43.9	1 (ref)		
	CT	56/58	50.9	1.44	0.88 to 2.34	0.149
	TT	12/27	69.2	3.09	1.72 to 5.55	**0.001**
MMP13: rs2252070	AA	42/33	44.0	1 (ref)		
	AG	63/57	47.5	1.29	0.77 to 2.16	0.334
	GG	19/38	66.7	2.02	1.13 to 3.60	**0.017**

SNP, single nucleotide polymorphism; MMP, matrix metalloproteases; HR, hazard ratio; ref, reference; OS, overall survival.

^#^ Adjusted for age, gender, histologic grade, gross type, tumor location, TNM stage and chemotherapy regimens. ^*^p<0.05 was considered significant and were depicted in bold. HR and P values were obtained by multivariate Cox Proportional analysis.

### Cumulative effects of two unfavorable SNPs on survival

**Figure 2:**
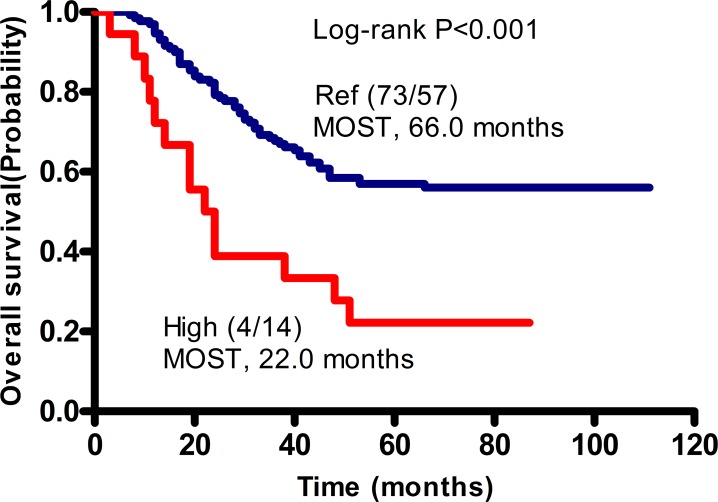
Cumulative effects of unfavorable SNPs on the median overall survival time (MOST) in patients with GA Kaplan-Meier analysis was performed. Patients without unfavorable genotype, namely the CT genotype of MMP2 rs2285053 and the CC or CT genotype of MMP8 rs1940475 were arbitrarily set as reference control (Ref). Patients with combined unfavorable genotypes containing CC of MMP2 rs2285053 and TT of MMP8 rs1940475 had a significantly shorten MOST and therefore were defined as high risk group (High). In parentheses, numerator represents the number of patients alive and denominator represents the number of patients died. P values were obtained by Log-rank test.

We showed that the co-presence of unfavorable SNPs in the *MMP2 rs2285053* CC and *MMP8 rs1940475* TT genotypes were associated with increased recurrence. Similarly, the mortality and death risk were significantly increased in patients with the combined *MMP2 rs2285053* CC and *MMP8 rs1940475* TT genotypes (death rate: 77.8%, OS: HR, 6.59; 95% CI, 3.15–13.79; p<0.0001) (Table 6). Kaplan–Meier analysis of patients with those combined genotypes showed that their median overall survival time (MOST) was 22 months, which was significantly shorter than that in patients with the *MMP2 rs2285053* CT and *MMP8 rs1940475* CC/CT genotypes (66 months, log rank p<0.001) (Figure 2).

**Table 6:** Cumulative effect of multiple SNPs and risk for survival

Group	Survival Alive (n)/Death (n)	Death rate (%)	OS		
			HR^#^	95% CI	P^*^
Reference**^§^**	73/57	43.8	1 (ref)		
High risk**^§^**	4/14	77.8	6.59	3.15 to 13.79	**<0.0001**

SNP, single nucleotide polymorphism; HR, hazard ratio; ref, reference; OS, overall survival.

^#^ Adjusted for age, gender, histologic grade, gross type, tumor location, TNM stage and chemotherapy regimens. ^*^p<0.05 was considered significant and were depicted in bold. HR and P values were obtained by multivariate Cox Proportional analysis.

^§^Reference: harbing CT genotype of MMP2 rs2285053 and CC/CT genotype of MMP8 rs1940475. ^§^High risk: the co-presence of CC genotype of MMP2 rs2285053 and TT genotype of MMP8 rs1940475.

### Effects of SNPs in *MMP2, 3*, and *8* on recurrence and survival

**Figure 3:**
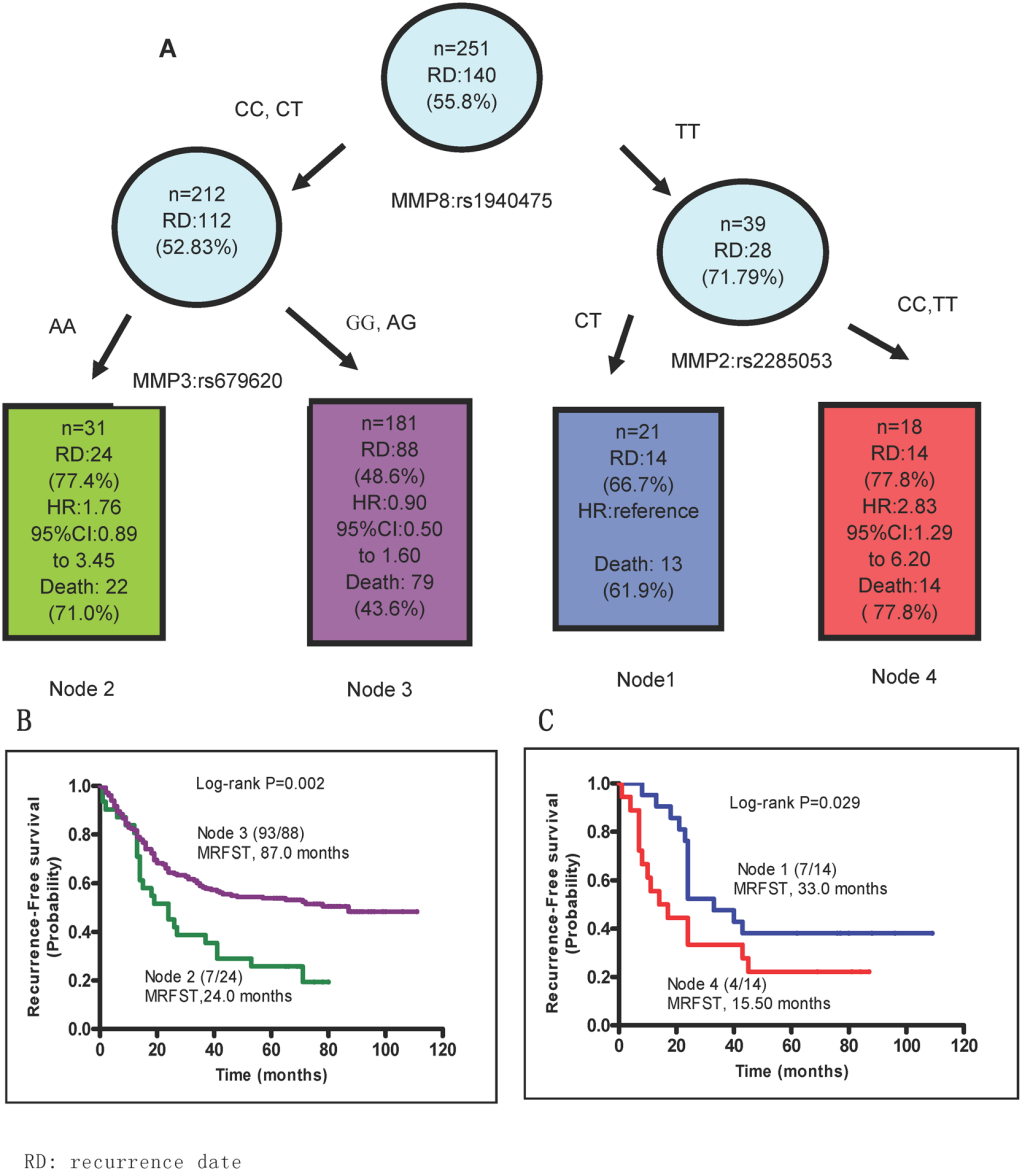
SNP-SNP interactions Patients with different combination of SNPs of *MMP8* rs1940475, *MMP3* rs679620 and *MMP2* rs2285053 SNPs were analyzed using a survival tree software and were split into 4 nodes. **(A)** SNP genotypes in each node are shown. Recurrence and patient death each node are presented. Patients in node 1, namely the TT genotype of MMP8 rs1940475 and CT genotype of MMP2 rs2285053 were set as the reference for the analysis of hazard ratio of recurrence risk. (**B** and **C**) Kaplan-Meier curves predict median recurrence-free survival time (MRFST) of each node.

After demonstrating that individual SNPs in *MMP2, 3*, and *8* may contribute to recurrence and survival in patients with GA, we examined the outcomes of these interactions. Using survival tree software developed by Zhang [15], the correlation between genotype interactions and MRFST was calculated. All likely combinations of two SNPs from *MMP2*, *3,* and *8* were tested. Patients were divided into four groups as described in Table 7. Patients with the combined *MMP8 rs1940475* TT and *MMP2 rs2285053* CT genotypes were arbitrarily defined as the basic group, namely Node 1, because these patients exhibited a medium recurrence rate (66.7%) with a MRFST of 33 months. Node 4, which had the combined *MMP8 rs1940475* TT and *MMP2 rs2285053* CC showed the poorest MRFST (15.5 months). MRFST was reduced to 24 months in Node 2 patients with the combined *MMP8 rs1940475* CC or CT genotype and *MMP3* rs679620 AA genotype. By contrast, the longest MRFST (87 months) and lowest recurrence rate (48.6%) were observed in Node 3 patients, who had the combined *MMP3 rs679620* GG or AG genotype and *MMP8 rs1940475* CC or CT genotype. Similarly, the lowest death rate was observed in Node 3 (43.6%) (Figure 3). Overall, the results of the survival tree analysis were consistent with our other data, and demonstrate an association between harboring a combination of unfavorable SNPs in *MMP2* and *MMP8* and a worse clinical outcome in GA patients.

**Table 7:** Survival tree nodes

Groups	MMP8 rs1940475	MMP3 rs679620	MMP2 rs2285053	Recurrence rate (%)	Death rate (%)
Node 1	TT		CT	66.7	61.9
Node 2	CC or CT	AA		77.4	71.0
Node 3	CC or CT	AG or GG		48.6	43.6
Node 4	TT		CC or TT	77.8	77.8

SNP, single nucleotide polymorphismterminal ; Node, terminal node.

### SNPs and *MMP2, 3* and *8* enzymatic activities

**Figure 4:**
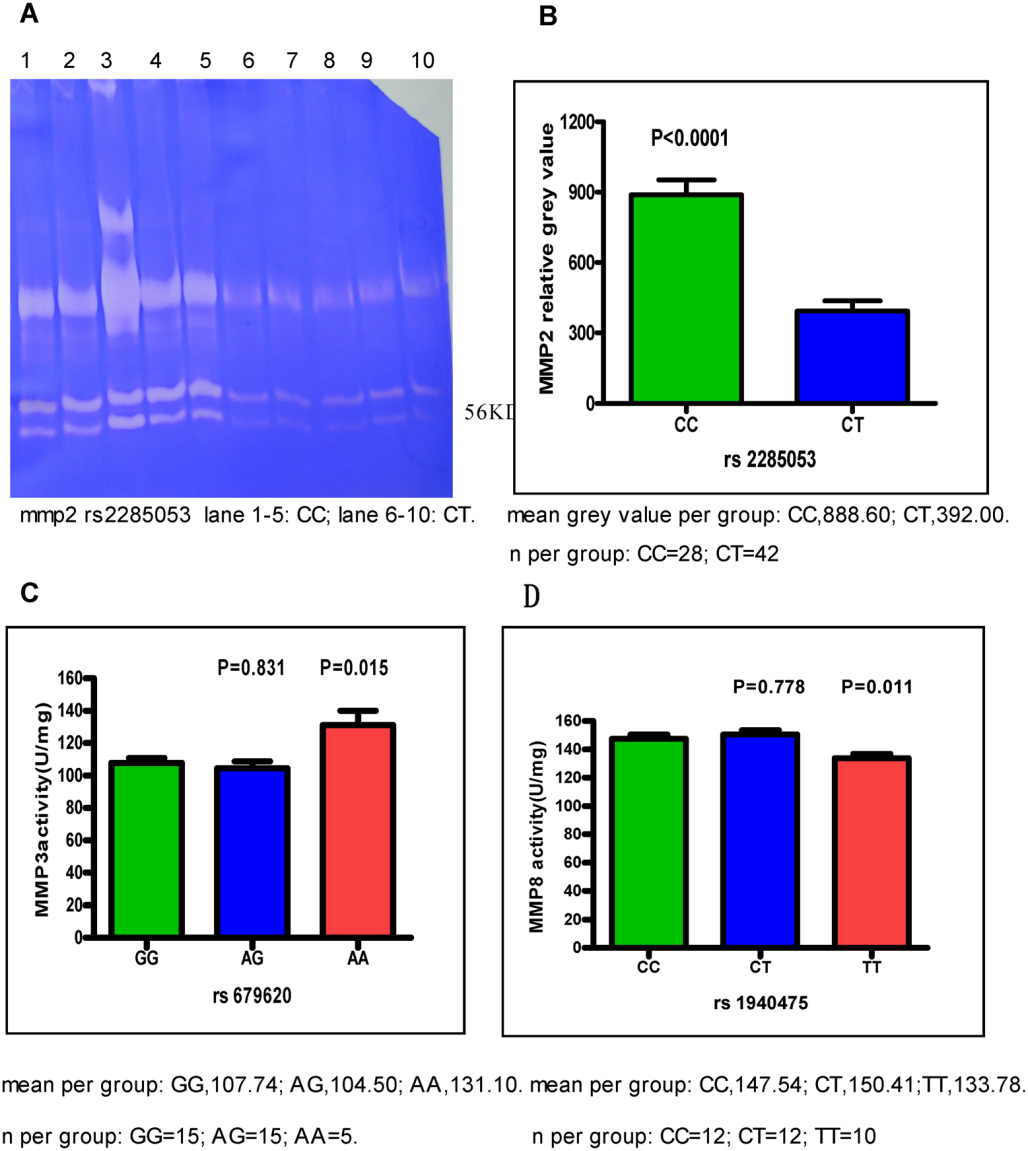
The activities of MMP2, MMP3 and MMP8 Cancer tissues were obtained from patients with CC and CT genotype of MMP2 rs2285053, patients with AA, GG, and AG genotype of MMP3 rs679620 and patients with CC, CT, and TT genotype of MMP8 rs1940475. Data were expressed as mean±SEM. **(A)** The representative gelatin gel of MMP2. CC genotype of MMP2 rs2285053 showed the highest MMP2 activities. Note that the lower molecular weight of 56 kD in MMP2 is active form. **(B)** MMP2 relative activity. Band intensity in gelatin gel was scanned and quantitated. CC vs CT, p<0.0001. **(C)** MMP3 activity. AG vs. GG, p=0.831; AA vs. GG, p=0.015. **(D)** MMP8 activity. CT vs. CC, p=0.778; TT vs. CC, p=0.011.

The enzymatic activities of different SNPs in *MMP2*, *3*, and *8* were examined. The gelatinase activities of MMP2 were measured via classical zymography, and were visibly increased in patients with the *MMP2 rs2285053* CC genotype (Figure 4A). The intensity of MMP2 bands was quantified by densitometry and found to be 888.60±64.00 (mean± standard error of mean (SEM)) in the CC genotype and 392.00±44.00 in the CT genotype (p<0.0001, Figure 4B). Among the *MMP3 rs679620* AA, AG, and GG genotypes, the highest activities were observed in the AA genotype (AA: 131.10±8.91 vs. GG: 107.74±3.11; p=0.015). The activities of MMP3 were comparable between the AA and AG genotypes (Figure 4C). In patients with the *MMP8 rs1940475* CC, CT, or TT genotype, high enzymatic activities were observed in CC (147.54±2.99) and CT (150.41±3.14) compared to TT (133.78±3.04, CC vs. TT, p=0.011) (Figure 4D, Supplementary Table 3).

### MMP2, 3 and 8 enzyme activities in normal and GA cancer tissues and MMP8 mRNA expression

MMP2 gelatinase activities in GA cancer tissues were visibly higher than those in normal tissues (Supplementary Figure 1A). The enzyme activities of MMP2 and 3 in GA cancer tissues were higher than those in normal tissues, and MMP8 enzyme activity was lower than those in normal tissues (MMP2, p=0.028; MMP3, p=0.012; MMP8, p=0.0029. Supplementary Figure 1B–1D). There was no statistically significant difference in the mRNA expression of different MMP8 *rs1940475* genotypes (p=0.915, Supplementary Table 4). The expression of MMP8 mRNA in GA cancer tissues was significantly lower than those in normal tissues (p=0.0048, Supplementary Figure 2).

### Association of SNPs with disease free survival (DFS)

Similar to RFS, patients with the *MMP2 rs2285053* CC genotype, *MMP3 rs679620* AA genotype or *MMP8 rs1940475* TT genotype also demonstrated poorer DFS (Supplementary Tables 5 and 6).

### Association of variables with RFS or OS with univariate and multivariate Cox Proportional analysis

Association of variables with RFS or OS was investigated by using univariate and multivariate Cox Proportional analysis, and the results were shown in Supplementary Tables 7 and 8.

## DISCUSSION

MMPs play vital roles in tumor pathology, especially in invasion and metastasis, due to their proteolysis of ECMs and effects on growth factors and angiogenesis [6, 16–18]. Abnormal MMP expression is frequently observed in cancers including GA [19]. In this study, we found an association between SNPs in *MMP2, 3,* and *8* with recurrence and death of GA patients. The MMP2 *rs2285053* CC, MMP8 *rs1940475* TT, and MMP3 *rs679620* AA genotypes were found to be correlated with increased GA recurrence and patient death. A computer-based survival tree analysis of these 254 GA patients indirectly validated the correlation of *MMP2*, *MMP8*, and *MMP3* with different clinical outcomes. Importantly, there was a significant cumulative effect of unfavorable *MMP2* and *MMP8* SNPs combination with clinical outcomes of GA.

*MMP2 rs2285053* is located −735 bp of the functional promoter region, with C and T as the two alleles in this SNP. The C allele is associated with the risk of lung cancer and nasopharyngeal carcinoma [20, 21]. In this study, we also found a correlation between the *MMP2 rs2285053* CC genotype with the risk of recurrence and survival in GA patients treated with fluoropyrimidine-based chemotherapy after operation. Importantly, the enzymatic assay revealed an increase in MMP2 degradation of the ECM in gastric cancer tissues obtained from patients with the *MMP2 rs2285053* CC genotype. Thus, the poor clinical outcome in patients with the CC genotype may directly correlate with increased MMP2 functional activities. It has been reported that approximately −735 bp of *MMP2 rs2285053* contains a SP1 transcription factor binding site that exhibits high affinity for the C allele. When C is replaced by T at −735, the binding to SP1 is significantly reduced, resulting in decreased MMP2 mRNA expression, and subsequently, less MMP2 protein expression and activity [22]. These data clearly explain the molecular basis of increased MMP2 activity in patients with the *MMP2 rs2285053* CC genotype.

*MMP3 rs679620* is located in exon 2 at the 45 amino acid position of the coding region. A missense mutation is generated with the G allele encoding glutamic acid and the A allele encoding lysine. The full-length protein of MMP3 in humans is 477 amino acids, with position 18 to 99 being the propeptide, position 100 to 477 as the enzymatic active sequence, and position 25 to 87 is a putative peptidoglycan binding region. Although the function of the peptidoglycan-binding region in MMPs is not clear, it may participate in the structure–function regulation of MMPs. Thus, different amino acids at position 45 of the peptidoglycan-binding region in MMP3 may result in difference in enzymatic activity; for example, lysine 45 encoded by the A allele leads to higher MMP3 activity, whereas glutamic acid 45 encoded by the G allele may lead to a corresponding reduction. Increased MMP3 activities may contribute to cancer progression.

We found that the *MMP8 rs1940475* TT genotype was associated with an increased risk of recurrence and death. *MMP8 rs1940475* is located in the propeptide coding region of exon 2 at the 87 amino acid position on chromosome 11q22. The T or C allele of this SNP is encodes for tyrosine and histidine respectively; thus, this allele is a missense mutation for MMP8. The full-length human MMP8 consists of 467 amino acids containing pro-peptide, catalytic, and hemopexin-like domains. During MMP8 activation, the N-terminal pro-peptide (106 amino acids) is removed by proteinases [23]. The function of the pro-peptide is to shield the zinc-binding region in the catalytic domain from water, to keep the enzyme in an inactive state [23]. We speculate that different amino acids at position 87 of the N-terminal, immediately next to the peptidoglycan-binding region, may affect the pro-protein structure of MMP8. The basic amino acid histidine encoded by the C allele of *MMP8 rs1940475* may increase the binding affinity of pro-protein to proteinases, whereas the neutral amino acid tyrosine encoded by the T allele has no such effect. This may explain the increased MMP8 activity in patients with the *MMP8 rs1940475* CC allele. In contrast to MMP2, the inhibitory effects of MMP8 on tumorigenesis and metastasis have been proposed based on several association studies, which showed that increased MMP8 activity was correlated with less metastasis and increased survival. For instance, Decock *et al.* [24, 25] reported that breast cancer patients with high MMP8 levels had low lymph node metastasis. Palavalli *et al.* [26, 27] showed that wild-type MMP8, and not the mutant, inhibited human melanoma cell growth and tumor formation. Consistent with these data, we observed an increase in cancer recurrence and death in GA patients with the *MMP8 rs1940475* TT allele and an associated decrease in MMP8 activity. We also found that different MMP8 rs1940475 genotypes did not result in statistically significant differences in mRNA expression levels.

In summary, this study reported for the first time the association of *MMP2 rs2285053*, *MMP8 rs1940475*, and *MMP3 rs679620* SNPs with poor clinical outcomes in patients with GA. The unfavorable effects of these SNPs on clinical outcomes may directly result from their structural–functional differences. Additional studies with a larger patient population are needed to confirm this correlation.

## MATERIALS AND METHODS

### Study population and treatment

A total of 254 GA patients with resectable adenocarcinoma and who underwent post-operative chemotherapy were prospectively recruited between 2006 and 2011 at Fujian Provincial Hospital. The follow-up period ended in March, 2016. The inclusion criteria were: age ≥18 years, received partial or total gastrectomy without major surgical complication(s), no history of other malignant tumors 5 years prior to gastric cancer, and no serious clinical infection(s). Chemotherapy was prescribed according to the National Comprehensive Cancer Network guideline. All patients were treated with two to five cycles of chemotherapy.

### Clinical data collection

Tumor-node-metastasis (TNM) stage based on clinical information and histopathologic examination was determined in patients using the American Joint Commission on Cancer Staging Manual. Patients were followed up every 6 months and the check-up included physical examination, blood work (particularly for serum tumor markers carcinoembryonic antigen, cancer antigen 125 [CA125], CA19-9, and CA724), abdominal ultrasonography, and chest x-ray or chest computed tomography. Gastric endoscopy was performed once a year. Computed tomography or magnetic resonance imaging was ordered if there was suspicion of metastasis. The diagnosis of recurrence was based on imaging studies, endoscopy, biopsy, or surgery. The mean follow-up time was 58 months. The study was approved by the ethics committee of Fujian Provincial Hospital. Investigators were blinded to the patient's genotype status.

### SNP selection and genotyping

First, potentially functional SNPs in *MMP1, 2, 3, 7, 8, 9, 12* and *13* were selected based on the following criteria: have potential functions (available at http://snpinfo.niehs.nih.gov/ [access June, 2013]); located at a conventional promoter, exon, or regulatory region that can directly affect gene expression and that encodes an amino acid; and tagged SNP with calculated r2 values ≥0.80 and minor-allele frequencies ≥0.05 in the Chinese Han population in the dbSNP or HapMap Project database, indicating that the SNP is common. Genotype data on each of MMP gene was found in the National Institute of Environmental Health Sciences (NIEHS) SNP database (available at http://snpinfo.niehs.nih.gov/ [access June, 2013]). Linkage disequilibrium between SNPs on the same chromosome was analyzed using the NIEHS SNP database, with an r^2^ > 0.8 considered as a high probability of haplotypes (available at http://snpinfo.niehs.nih.gov/ [access June, 2013]). Additionally, SNPs associated with other cancers reported in previous studies [13, 28–33] (Supplementary Table 1) were selected. As a result, we selected 10 SNPs for this study: *MMP1 rs1799750* (5′ untranslated region), *MMP2 rs2285053* and *rs243865* (promoter region), *MMP3 rs679620* (promoter region), *MMP7 rs11568818* (promoter region), *MMP8 rs1940475* (exon 2), *MMP9 rs17576* and *rs2250889* (promoter region), *MMP12 rs2276109* (promoter region), and *MMP13 rs2252070* (promoter region) (Table 2, Supplementary Table 1). Finally, the allele frequency of SNPs in 254 patients needed to pass the Hardy–Weinberg equilibrium test, as allele frequency that was significantly different from the reported frequency in the general Han population was likely an underrepresentation (p<0.05). Thus, we excluded *MMP7 rs11568818* and *MMP12 rs2276109* from further analyses. Genomic DNA from 254 fresh cancer tissues was extracted using the QIAamp DNA Kit (Qiagen GmbH, Hilden, Germany). The standard genotyping protocol was followed using the Sequenom's MassARRAY system. Quality control measures were applied at each step. Overall, the genotyping error rate was less than 0.1%.

### MMP2, 3, and 8 activities in tissues and MMP8 mRNA expression

Fresh GA cancer and paired normal tissues were snap-frozen in liquid nitrogen. Tissue proteins were extracted after homogenization, and MMP2 activity was measured by zymography as previously described [34]. The gels were stained with Coomassie blue, and band density was determined using Quantity One 4.62. Since MMP3 and MMP8 were not gelatinase, their activities were determined by fluorescence resonance energy transfer and the colorimetric method [35, 36]. Recombinant MMP3 and MMP8 proteins were used as positive controls and for activity calculations. MMP8 mRNA expression was determined using the Promega qRT-PCR Reagent Kit (Promega Corporation, Madison, WI, USA). The 5′ to 3′ primer sequence was as follows: GAPDH (forward, GAGTCAACGGATTTGGTC GT; reverse, TTGATTTTGGAGGGATCTCG); MMP8 (forward AAAACTGTTCA GGACTACCTGG; reverse, ATTTGGCTTCCCCGTCACAT).

### Statistical analysis

The relationship between patient characteristics and survival/recurrence was analyzed using the Pearson Chi-squared test or the Fisher's exact test. HRs between SNPs and overall survival (OS) or recurrence-free survival (RFS) times were calculated using the COX model after adjusting for age, gender, histologic grade, gross examination results, tumor location, TNM stage and Chemotherapy regimens. The log-rank tests and Kaplan-Meier curve were used to evaluate differences in recurrence-free and overall survival times. Additionally, 95% CIs were calculated. Finally, the cumulative effects of two unfavorable genotypes were assessed from the main effect of analysis of single SNP [37]. All statistical analyses were performed using the SPSS software version 17.0 (SPSS Inc., Chicago, IL). Specifically, the interactions among SNPs and the interactions on recurrence and survival were analyzed using TREE software (available at http://C2S2.yale.edu/software/stree/) [access June, 2013], which utilizes recursive-partitioning to confirm subgroups of individuals at higher risk of disease recurrence [37]. The principle and practical guide of tree analysis including splitting criteria and tree pruning rules have been extensively described by Zhang and Singer [15]. The paired *t-*test or one-way analysis of variance was used to analyze MMP2, 3, and 8 enzymatic activities and MMP8 mRNA expression. P values less than 0.05 were considered statistically significant.

## SUPPLEMENTARY MATERIALS FIGURES AND TABLES





## Abbreviations

MMPs: matrix metalloproteinases
SNP: single nucleotide polymorphisms
GA: gastric adenocarcinoma
HR: hazard ratio
CI: confidence interval
MOST: median overall survival time
MRFST: median recurrence free survival time
SEM: standard error of mean
OS: overall survival
RFS: recurrence-free survival
