# Supplementary material for: Polymorphisms in matrix metalloproteinases 2, 3, and 8 increase recurrence and mortality risk by regulating enzyme activity in gastric adenocarcinoma
Source: Oncotarget. 2017 Nov 20;8(62):105971–83. doi: 10.18632/oncotarget.22516 (PMC5739694; doi:10.18632/oncotarget.22516)
Supplement: Supplementary file 1 [file oncotarget-08-105971-s001.pdf]

Polymorphisms in matrix metalloproteinases 2, 3, and 8 increase recurrence and mortality risk by regulating enzyme activity in gastric adenocarcinoma

SUPPLEMENTARY MATERIALS

A

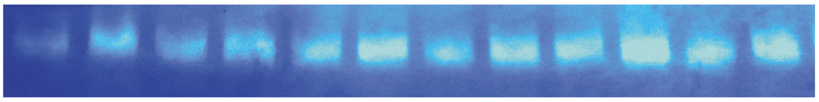

N1 C1 N2 C2 N3 C3 N4 C4 N5 C5 N6 C6

mmp2 enzymatic activity, N: normal tissue; C: cancer tissue

B

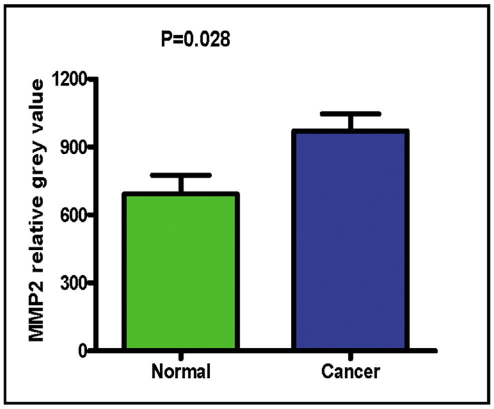

mean grey value per group: Normal, 692; Cancer, 972.

n=12

C

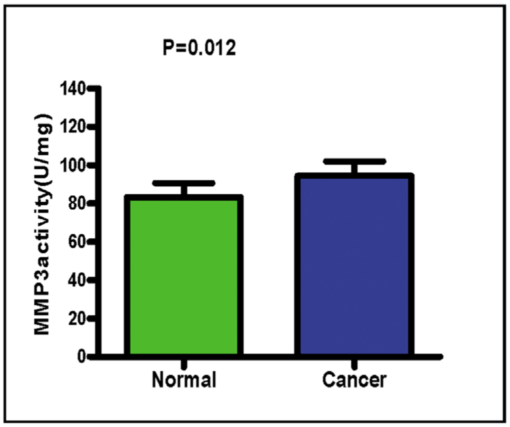

mean per group: Normal, 83.18; Cancer, 94.38.

n=12

D

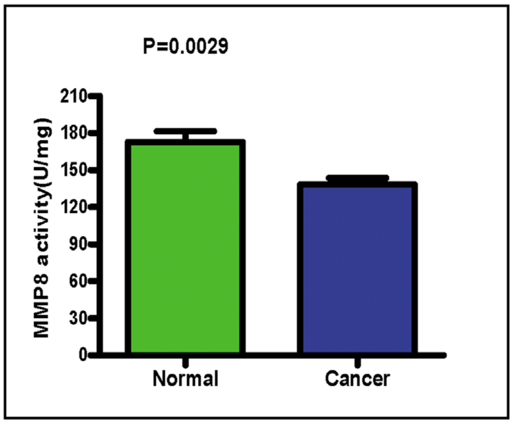

mean per group: Normal, 172.80; Cancer, 138.30.

n=12

Supplementary Figure 1: The activities of MMP2, 3 and 8 in normal and GA cancer tissues. Data were expressed as mean±SEM. **(A)** The representative gelatin gel of MMP2. Cancer tissues showed the higher MMP2 activities. **(B)** MMP2 relative activity. Band intensity in gelatin gel was scanned and quantified (P=0.028). **(C)** MMP3 activity. p=0.012. **(D)** MMP8 activity. p=0.0029.

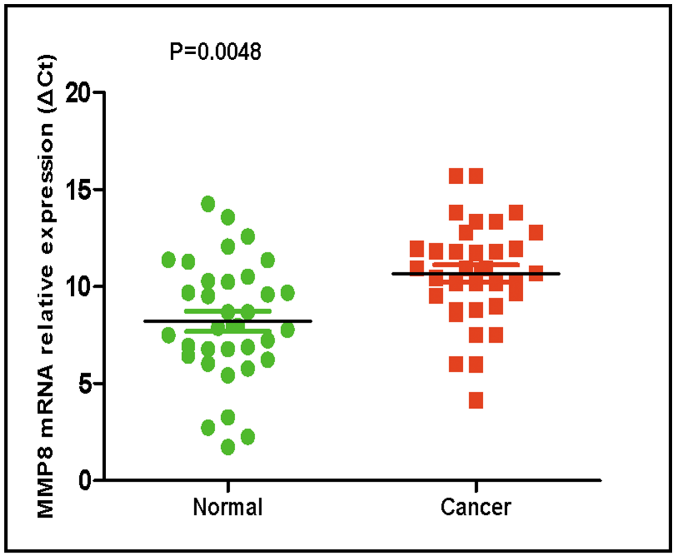

ΔCt= mmp8 Ct- GAPDH Ct

mean per group:Normal,8.20; Cancer, 10.66. n=34

Supplementary Figure 2: MMP8 mRNA relative expession in normal and cancer tissues. Data were expressed as mean±SEM.

Supplementary Table 1: The ten SNPs information and previous study

Gene	Tag SNPS number	Current SNP ID	Base changes	Position in gene	Region in gene	Amino acid changes	Previous study about genotype and diseases	References number
MMP1	68	rs1799750	1G →2G	-1607bp	5' untranslated region		2G increases the risk of renal cell carcinoma	26
MMP2	60	rs2285053	C→T	-735bp	promoter		CC promotes occurrence and metastasis of esophageal cancer by increase the binding of Sp1 and displays a higher promoter activity in esophageal cancer, which is examined by electrophoretic mobility shift assays, luciferase gene expression assays, and reverse transcriptase-PCR analyses.	21
MMP2	60	rs243865	C→T	-1306bp	promoter		CT+TT increase the risk of gallbladder cancer	27
MMP3	48	rs679620	A→G	5723bp	exon 2	missense, lysine → glutamic	AA promote the development of periapical lesions	30
MMP7	62	rs11568818	A→G	-181bp	promoter		AG+GG increase the risk of gallbladder cancer	27
MMP8	109	rs1940475	C→T	7438bp	exon 2	missense, histidine → tyrosine	GG promotes the lymph node metastasis in breast cancer	22
MMP9	41	rs17556	G→T	-279bp	promoter		GT+TT increase the risk of gallbladder cancer	27
MMP9	41	rs2250889	C→G	-574bp	promoter		CG+GG increase the Susceptibility to lung cancer	31
MMP12	60	rs2276109	A→G	-82bp	promoter		no significant association SNP with breast cancer AA is connected with a higher risk of disseminated colorectal cancer	13 32
MMP13	63	rs2252070	A→G	-77bp	promoter		no significant association SNP with breast cancer no significant association SNP with disseminated colorectal cancer	13 32

The numbers of tag SNPs which had r2 values ≥0.80 and minor-allele frequencies (MAFs) ≥0.05 and located within 10 kb upstream of the transcriptional start site and 10 kb downstream of the transcriptional stop site were obtained from CHB population in dbSNP datebase.

Supplementary Table 2: SNPs and patients characteristics

	MMP2 rs2285053		MMP3 rs679620		MMP8 rs1940475		MMP13 rs2252070	
	N(CT/CC/TT)	P	N(GG/AG/AA)	P	N(CC/CT/TT)	P	N(AA/AG/GG)	P
**Age**		0.800		0.451		0.969		0.083
<60	91/53/9		72/57/24		60/68/24		41/81/30	
≧60	60/33/8		44/45/12		38/46/15		34/39/27	
**Gender**		0.940		0.727		0.091		0.132
Male	110/61/12		84/75/24		67/89/24		60/80/41	
Female	41/25/5		32/27/12		31/25/15		15/40/16	
**Histologic grade**		0.225		0.988		0.603		0.855
WD	7/6/3		7/7/2		6/5/4		6/7/2	
MD	99/51/8		72/63/23		63/72/21		47/75/35	
PD	45/29/6		37/32/11		29/37/14		22/38/20	
**Gross type**		0.677		0.509		0.434		0.477
Superficial	2/1/1		2/2/0		1/2/1		1/3/0	
Apophysis	10/7/1		6/11/1		6/8/4		8/7/3	
Invasion	138/78/15		107/89/35		91/104/33		65/110/54	
Massive type	1/0/0		1/0/0		0/0/1		1/0/0	
**Tumor location**		0.728		0.757		0.067		0.076
Cardiac	19/13/3		13/7/5		6/24/5		8/16/10	
Gastric fundus	2/2/0		2/1/1		2/2/0		2/11/1	
Gastric body	10/2/0		6/6/0		7/3/2		0/11/1	
Gastric antrum	118/66/14		93/76/29		81/82/32		63/90/44	
Whole stomach	2/3/0		2/2/1		2/3/0		2/2/1	
**Chemotherapy**		0.818		0.619		0.169		0.927
Fuoropyrimidine only	46/24/6		35/28/13		24/41/10		22/37/16	
Fuoropyrimidine +Platinum	105/62/11		81/74/23		74/73/29		53/83/41	
**TNM stage**		0.604		0.555		0.323		0.572
1+2	74/46/7		54/55/18		43/61/21		41/59/26	
3+4	77/40/10		62/47/18		55/53/18		34/61/31	

Supplementary Table 3: Statistical results of MMP8 activity

rs1540479 genotype	MMP8 activity					
	N	Mean	SD	SEM	Minimum value	Maximum value
CC	12	147.54	10.36	2.99	121.20	160.20
CT	12	150.41	10.88	3.14	125.10	165.82
TT	10	133.78	9.60	3.04	117.40	148.20

Supplementary Table 4: MMP8 rs1940475 genotype and mRNA downexpession

rs 1940475 genotype	n	Downexpression		P#
		Yes/No (n)	%	
CC	12	6/6	50.0	0.915
CT	12	7/5	58.3	
TT	10	6/4	60.0	

MMP8 mRNA downexpession: Yes, down change fold ≧2.0; No, down change fold <2.0.
#: Fisher exact test.

Supplementary Table 5: Patients characteristics and DFS

Characteristics	n (N=254)	%	N (No/Yes)	P*
**Age**				0.422
< 65	193	76	84 / 109	
≥65	61	24	23 /38	
**Gender**				0.268
Male	183	72.0	81 /102	
Female	71	28.0	26 / 45	
**Histologic grade**				0.436
Well differentiated	16	6.3	7 / 9	
Moderately differentiated	158	62.2	71 / 87	
Poorly differentiated	80	31.5	29 / 51	
**Gross type**				0.276
Superficial	4	1.6	3/1	
Apophysis	18	7.1	9 / 9	
Invasion	231	90.9	94 / 137	
Massive type	1	0.4	1 / 0	
**Tumor location**				0.527
Cardiac	35	13.8	12 / 23	
Gastric fundus	4	1.6	2 / 2	
Gastric body	12	4.7	7 / 5	
Gastric antrum	198	78.0	85/ 113	
Whole stomach	5	2.0	1 / 4	
**Chemotherapy**				0.164
Fuoropyrimidine only	76	29.9	27/49	
Fuoropyrimidine+Platinum	178	70.1	80/98	
**DFS# (No/Yes)**	107 / 147	42.1 / 57.9		
**TNM stage**				**<0.001**
**1**	54	21.3	41/13	
**2**	73	28.7	29/44	
**3**	111	43.7	35/76	
**4**	16	6.3	2/14	

DFS, disease free survival. *p<0.05 was considered significant and are depicted in bold.

Supplementary Table 6: Variables and DFS with univariate and multivariate analysis

See Supplementary File 1

Supplementary Table 7: Variables and RFS with univariate and multivariate analysis

See Supplementary File 1

Supplementary Table 8: Variables and OS with univariate and multivariate analysis

See Supplementary File 1
