# Supplementary material for: Polymorphisms in matrix metalloproteinases 2, 3, and 8 increase recurrence and mortality risk by regulating enzyme activity in gastric adenocarcinoma
Source: Oncotarget. 2017 Nov 20;8(62):105971–83. doi: 10.18632/oncotarget.22516 (PMC5739694; doi:10.18632/oncotarget.22516)
Supplement: Supplementary file 2 [file oncotarget-08-105971-s002.docx]

| **Supplementary Table 6: Variables and DFS with univariate and multivariate analysis** | | | | | | | |
| --- | --- | --- | --- | --- | --- | --- | --- |
| **Variable** | | **Univariate analysis** | | | **Multivariate analysis** | | |
|  |  | **HR^#^** | **95% CI** | **P*** | **HR^#^** | **95% CI** | **P*** |
| **Gender** | **Male** | 1 (ref) |  |  | 1 (ref) |  |  |
|  | **Female** | 1.16 | 0.82 to 1.67 | 0.406 | 1.00 | 0.65 to 1.54 | 0.996 |
| **Age (years)** | **< 65** | 1 (ref) |  |  | 1 (ref) |  |  |
|  | **≥65** | 1.18 | 0.82 to 1.71 | 0.380 | 1.52 | 0.95 to 2.44 | 0.081 |
| **Histologic grade** | **Well differentiated** | 1 (ref) |  |  | 1 (ref) |  |  |
|  | **Moderately differentiated** | 1.09 | 0.55 to 2.18 | 0.800 | 1.62 | 0.58 to 4.48 | 0.356 |
|  | **Poorly differentiated** | 1.38 | 0.68 to 2.81 | 0.379 | 1.92 | 0.69 to 5.38 | 0.213 |
| **Gross type** | **Superficial** | 1 (ref) |  |  | 1 (ref) |  |  |
|  | **Apophysis** | 2.85 | 0.36 to 22.49 | 0.321 | 1.20 | 0.14 to 10.54 | 0.869 |
|  | **Invasion** | 3.45 | 0.48 to 24.66 | 0.218 | 1.19 | 0..15 to 9.20 | 0.869 |
|  | **Massive type** | 0.00 | 0.00 to 830.00 | 0.959 | 0.00 | 0.00 to218.00 | 0.959 |
| **Tumor location** | **Cardiac** | 1 (ref) |  |  | 1 (ref) |  |  |
|  | **Gastric fundus** | 0.91 | 0.21 to 3.84 | 0.892 | 1.00 | 0.20 to 5.11 | 0.999 |
|  | **Gastric body** | 0.49 | 0.19 to1.29 | 0.150 | 0.47 | 0.15 to 1.49 | 0.200 |
|  | **Gastric antrum** | 0.83 | 0.53 to 1.29 | 0.402 | 0.96 | 0.56 to 1.63 | 0.865 |
|  | **Whole stomach** | 1.71 | 0.59 to4.94 | 0.324 | 5.08 | 1.52 to 16.96 | **0.008** |
| **Chemotherapy** | **Fuoropyrimidine only** | 1 (ref) |  |  | 1 (ref) |  |  |
|  | **Fuoropyrimidine +Platinum** | 0.83 | 0.58 to 1.17 | 0.289 | 0.77 | 0.50 to 1.18 | 0.225 |
| **TNM stage** | **1** | 1 (ref) |  |  | 1 (ref) |  |  |
|  | **2** | 3.92 | 2.02 to 7.59 | **< 0.001** | 4.06 | 1.98 to 8.36 | **< 0.001** |
|  | **3** | 5.36 | 2.83 to10.13 | **< 0.001** | 5.97 | 3.01 to 11.84 | **< 0.001** |
|  | **4** | 10.85 | 4.88 to 24.13 | **< 0.001** | 13.92 | 5.48 to 35.31 | **< 0.001** |
| **MMP1:** | **1G/1G** | 1 (ref) |  |  | 1 (ref) |  |  |
| **rs1799750** | **1G/2G** | 1.09 | 0.65 to 1.82 | 0.751 | 1.92 | 0.90 to 4.09 | 0.092 |
|  | **2G/2G** | 1.10 | 0.65 to 1.87 | 0.720 | 1.87 | 0.79 to 4.44 | 0.156 |
| **MMP2:** | **CT** | 1 (ref) |  |  | 1 (ref) |  |  |
| **rs2285053** | **CC** | 1.59 | 1.03 to2.45 | **0.037** | 1.83 | 0.96 to 3.58 | **0.021** |
|  | **TT** | 2.67 | 1.28 to 5.59 | **0.009** | 3.05 | 1.15 to 5.04 | **0.013** |
| **MMP2:** | **CC** | 1 (ref) |  |  | 1 (ref) |  |  |
| **rs243865** | **CT** | 1.14 | 0.75 to 1.71 | 0.544 | 1.17 | 0.72 to 1.90 | 0.526 |
|  | **TT** | 0.39 | 0.05 to 2.79 | 0.347 | 0.49 | 0.06 to 4.41 | 0.527 |
| **MMP3:** | **GG** | 1 (ref) |  |  | 1 (ref) |  |  |
| **rs679620** | **AG** | 0.81 | 0.56 to 1.16 | 0.245 | 0.63 | 0.37 to 1.06 | 0.082 |
|  | **AA** | 1.71 | 0.97 to 3.35 | **0.041** | 2.02 | 0.87 to 3.96 | **0.046** |
| **MMP8:** | **CC** | 1 (ref) |  |  | 1 (ref) |  |  |
| **rs1940475** | **CT** | 1.13 | 0.78 to 1.64 | 0.514 | 1.12 | 0.73 to1.73 | 0.603 |
|  | **TT** | 1.81 | 0.94 to 2.90 | **0.025** | 2.60 | 1.06 to 4.05 | **0.011** |
| **MMP9:** | **GG** | 1 (ref) |  |  | 1 (ref) |  |  |
| **rs17556** | **GT** | 0.67 | 0.21 to 2.13 | 0.498 | 1.21 | 0.35 to 4.18 | 0.768 |
|  | **TT** | 1.84 | 0.46 to7.45 | 0.392 | 0.99 | 0.20 to 5.00 | 0.998 |
| **MMP9:** | **CC** | 1 (ref) |  |  | 1 (ref) |  |  |
| **rs2250889** | **CG** | 1.12 | 0.79 to 1.58 | 0.527 | 1.21 | 0.82 to 1.80 | 0.334 |
|  | **GG** | 1.06 | 0.51 to 2.19 | 0.874 | 1.34 | 0.58 to 3.10 | 0.495 |
| **MMP13:** | **AA** | 1 (ref) |  |  | 1 (ref) |  |  |
| **rs2252070** | **AG** | 1.07 | 0.72 to 1.59 | 0.738 | 1.35 | 0.84 to 2.18 | 0.216 |
|  | **GG** | 1.53 | 0.98 to 2.38 | 0.061 | 1.44 | 0.84 to 2.46 | 0.182 |
| DFS, disease free survival; MMP, matrix metalloproteases; HR, hazard ratio; ref, reference. | | | | | | | |
| # Adjusted for age, gender, histologic grade, gross type, tumor location, TNM stage and chemotherapy regimens. *p<0.05 was considered significant and were depicted in bold. HR and P values were obtained by multivariate Cox Proportional analysis. | | | | | | | |
|  |  |  |  |  |  |  |  |

| **Supplementary Table 7: Variables and RFS with univariate and multivariate analysis** | | | | | | | |
| --- | --- | --- | --- | --- | --- | --- | --- |
| **Variable** | | **Univariate analysis** | | | **Multivariate analysis** | | |
|  |  | **HR^#^** | **95% CI** | **P*** | **HR^#^** | **95% CI** | **P*** |
| **Gender** | **Male** | 1 (ref) |  |  | 1 (ref) |  |  |
|  | **Female** | 1.14 | 0.80 to 1.64 | 0.466 | 0.99 | 0.80 to 1.64 | 0.950 |
| **Age (years)** | **< 65** | 1 (ref) |  |  | 1 (ref) |  |  |
|  | **≥65** | 1.19 | 0.82 to 1.73 | 0.366 | 1.53 | 0.94 to 2.46 | 0.085 |
| **Histologic grade** | **Well differentiated** | 1 (ref) |  |  | 1 (ref) |  |  |
|  | **Moderately differentiated** | 1.06 | 0.53 to 2.12 | 0.861 | 1.55 | 0.55 to 4.34 | 0.405 |
|  | **Poorly differentiated** | 1.29 | 0.63 to 2.63 | 0.489 | 1.79 | 0.63 to 5.06 | 0.276 |
| **Gross type** | **Superficial** | 1 (ref) |  |  | 1 (ref) |  |  |
|  | **Apophysis** | 2.84 | 0.36 to22.43 | 0.322 | 1.05 | 0.12 to 9.38 | 0.966 |
|  | **Invasion** | 3.30 | 0.46 to 23.58 | 0.235 | 1.06 | 0..14 to 8.29 | 0.955 |
|  | **Massive type** | 0.00 | 0.00 to 253. 00 | 0.959 | 0.00 | 0.00 to 523.00 | 0.959 |
| **Tumor location** | **Cardiac** | 1 (ref) |  |  | 1 (ref) |  |  |
|  | **Gastric fundus** | 0.91 | 0.22 to 3.87 | 0.900 | 1.03 | 0.20 to 5.32 | 0.970 |
|  | **Gastric body** | 0.49 | 0.19 to 0.30 | 0.152 | 0.50 | 0.16 to 1.60 | 0.240 |
|  | **Gastric antrum** | 0.79 | 0.50 to 1.24 | 0.300 | 0.89 | 0.52 to 1.54 | 0.680 |
|  | **Whole stomach** | 0.59 | 0.59 to4.92 | 0.328 | 4.99 | 1.50 to 16.65 | **0.009** |
| **Chemotherapy** | **Fuoropyrimidine only** | 1 (ref) |  |  | 1 (ref) |  |  |
|  | **Fuoropyrimidine +Platinum** | 0.84 | 0.59 to 1.20 | 0.399 | 0.73 | 0.47 to 1.14 | 0.167 |
| **TNM stage** | **1** | 1 (ref) |  |  | 1 (ref) |  |  |
|  | **2** | 3.63 | 1.86 to 7.06 | **<0.001** | 4.02 | 1.88 to 8.62 | **<0.001** |
|  | **3** | 5.19 | 2.74to 9.82 | **<0.001** | 6.21 | 3.02 to 12.79 | **<0.001** |
|  | **4** | 10.78 | 4.84 to 23.95 | **<0.001** | 14.6 | 5.63 to 37.88 | **<0.001** |
| **MMP1:** | **1G/1G** | 1 (ref) |  |  |  |  |  |
| **rs1799750** | **1G/2G** | 1.04 | 0.62 to 1.74 | 0.020 | 2.09 | 0.96 to 4.53 | 0.062 |
|  | **2G/2G** | 1.08 | 0.64 to 1.84 | 0.090 | 1.95 | 0.82 to 4.67 | 0.134 |
| **MMP2:** | **CT** | 1 (ref) |  |  | 1 (ref) |  |  |
| **rs2285053** | **CC** | 2.32 | 0.93 to 3.89 | **0.004** | 1.85 | 1.19 to 2.85 | **0.006** |
|  | **TT** | 3.79 | 1.01 to 6.16 | **0.002** | 2.87 | 1.42 to 5.83 | **0.003** |
| **MMP2:** | **CC** | 1 (ref) |  |  | 1 (ref) |  |  |
| **rs243865** | **CT** | 1.19 | 0.79 to 1.79 | 0.414 | 1.24 | 0.76 to 2.02 | 0.386 |
|  | **TT** | 0.40 | 0.06 to 2.94 | 0.375 | 0.55 | 0.06 to 4.98 | 0.593 |
| **MMP3:** | **GG** | 1 (ref) |  |  | 1 (ref) |  |  |
| **rs679620** | **AG** | 0.72 | 0.52 to 1.10 | 0.137 | 0.72 | 0.42 to 1.23 | 0.233 |
|  | **AA** | 2.52 | 0.98 to 5.38 | **0.025** | 2.15 | 1.04 to 4.45 | **0.040** |
| **MMP8:** | **CC** | 1 (ref) |  |  | 1 (ref) |  |  |
| **rs1940475** | **CT** | 1.70 | 0.22 to 2.93 | 0.056 | 1.54 | 0.97 to 2.45 | 0.066 |
|  | **TT** | 2.93 | 0.48 to 5.96 | **0.003** | 2.22 | 1.27 to 3.87 | **0.005** |
| **MMP9:** | **GG** | 1 (ref) |  |  |  |  |  |
| **rs17556** | **GT** | 1.14 | 0.78 to 1.66 | 0.490 | 1.17 | 0.33 to 4016 | 0.806 |
|  | **TT** | 1.57 | 0.98 to 2.51 | 0.061 | 1.87 | 0.22 to 5.41 | 0.907 |
| **MMP9:** | **CC** | 1 (ref) |  |  |  |  |  |
| **rs2250889** | **CG** | 1.12 | 0.79 to 1.60 | 0.521 | 1.19 | 0.79 to 1.79 | 0.399 |
|  | **GG** | 1.10 | 0.53 to 2.28 | 0.794 | 1.35 | 0.58 to 3.14 | 0.488 |
| **MMP13:** | **AA** | 1 (ref) |  |  |  |  |  |
| **rs2252070** | **AG** | 1.09 | 0.73 to 1.64 | 0.663 | 1.33 | 0.82 to 2.18 | 0.251 |
|  | **GG** | 1.56 | 1.00 to 2.46 | 0.052 | 1.46 | 0.84 to 2.54 | 0.176 |
| RFS, recurrence free survival; MMP, matrix metalloproteases; HR, hazard ratio; ref, reference. | | | | | | | |
| # Adjusted for age, gender, histologic grade, gross type, tumor location, TNM stage and chemotherapy regimens. *p<0.05 was considered significant and were depicted in bold. HR and P values are obtained by multivariate Cox Proportional analysis. | | | | | | | |
|  |  |  |  |  |  |  |  |

| **Supplementary Table 8: Variables and OS with univariate and multivariate analysis** | | | | | | | |
| --- | --- | --- | --- | --- | --- | --- | --- |
| **Variable** | | **Univariate analysis** | | | **Multivariate analysis** | | |
|  |  | **HR^#^** | **95% CI** | **P*** | **HR^#^** | **95% CI** | **P*** |
| **Gender** | **Male** | 1 (ref) |  |  | 1 (ref) |  |  |
|  | **Female** | 1.08 | 0.74 to 1.57 | 0.703 | 0.99 | 0.63 to 1.55 | 0.947 |
| **Age (years)** | **< 65** | 1 (ref) |  |  | 1 (ref) |  |  |
|  | **≥65** | 1.31 | 0.89 to 1.92 | 0.174 | 1.53 | 1.06 to 2.61 | 0.057 |
| **Histologic grade** | **Well differentiated** | 1 (ref) |  |  | 1 (ref) |  |  |
|  | **Moderately differentiated** | 0.82 | 0.41 to 1.64 | 0.573 | 0.90 | 0.32 to2.51 | 0.840 |
|  | **Poorly differentiated** | 1.10 | 0.54 to 2.24 | 0.801 | 1.12 | 0.39 to 3.22 | 0.827 |
| **Gross type** | **Superficial** | 1 (ref) |  |  | 1 (ref) |  |  |
|  | **Apophysis** | 198.45 | 0.00 to 2530.80 | 0.912 | 78.71 | 0.00 to 1194.80 | 0.925 |
|  | **Invasion** | 233.56 | 0.00 to 2974.80 | 0.910 | 77.89 | 0.00 to 1179.85 | 0.925 |
|  | **Massive type** | 1.00 | 0.00 to 619.17 | 1.000 | 0.21 | 0.00 to 552.20 | 0.995 |
| **Tumor location** | **Cardiac** | 1 (ref) |  |  | 1 (ref) |  |  |
|  | **Gastric fundus** | 1.13 | 0.26 to 4.84 | 0.869 | 1.67 | 0.34 to 8.22 | 0.529 |
|  | **Gastric body** | 0.34 | 0.10 to 1.13 | 0.079 | 0.25 | 0.05 to 1.17 | 0.078 |
|  | **Gastric antrum** | 0.87 | 0.54 to 1.41 | 0.581 | 0.91 | 0.51 to 1.62 | 0.750 |
|  | **Whole stomach** | 0.69 | 0.16 to2.97 | 0.621 | 3.20 | 0.65 to 15.71 | 0.151 |
| **Chemotherapy** | **Fuoropyrimidine only** | 1 (ref) |  |  | 1 (ref) |  |  |
|  | **Fuoropyrimidine +Platinum** | 0.74 | 0.51 to 1.06 | 0.104 | 0.70 | 0.45 to 1.10 | 0.124 |
| **TNM stage** | **1** | 1 (ref) |  |  | 1 (ref) |  |  |
|  | **2** | 6.52 | 2.46 to 15.37 | **<0.001** | 7.10 | 2.76 to 18.29 | **<0.001** |
|  | **3** | 8.50 | 3.69 to 15.59 | **<0.001** | 10.31 | 4.15 to 25.58 | **<0.001** |
|  | **4** | 15.70 | 5.95 to 41.46 | **<0.001** | 17.33 | 5.64 to 53.29 | **<0.001** |
| **MMP1:** | **1G/1G** | 1 (ref) |  |  |  |  |  |
| **rs1799750** | **1G/2G** | 1.03 | 0.60 to 1.77 | 0.910 | 1.94 | 0.87 to 4.34 | 0.104 |
|  | **2G/2G** | 1.03 | 0.59 to 1.79 | 0.922 | 1.64 | 0.65 to 4.14 | 0.291 |
| **MMP2:** | **CT** | 1 (ref) |  |  | 1 (ref) |  |  |
| **rs2285053** | **CC** | 2.45 | 1.01 to 5.09 | **0.002** | 2.03 | 1.29 to 3.20 | **0.002** |
|  | **TT** | 1.95 | 0.57 to 4.30 | 0.098 | 1.77 | 0.81 to 3.89 | 0.152 |
| **MMP2:** | **CC** | 1 (ref) |  |  | 1 (ref) |  |  |
| **rs243865** | **CT** | 1.10 | 0.72 to 1.69 | 0.670 | 1.13 | 0.68 to 1.89 | 0.637 |
|  | **TT** | 0.46 | 0.06 to 3.28 | 0.437 | 0.84 | 0.10 to 7.48 | 0.877 |
| **MMP3:** | **GG** | 1 (ref) |  |  | 1 (ref) |  |  |
| **rs679620** | **AG** | 0.77 | 0.53 to 1.14 | 0.195 | 0.83 | 0.13 to 0.57 | 0.516 |
|  | **AA** | 3.47 | 0.92 to 8.34 | **0.001** | 3.25 | 1.50 to 7.02 | **0.003** |
| **MMP8:** | **CC** | 1 (ref) |  |  | 1 (ref) |  |  |
| **rs1940475** | **CT** | 1.15 | 0.77 to 1.70 | 0.193 | 1.44 | 0.88 to 2.34 | 0.149 |
|  | **TT** | 2.95 | 1.20 to 5.01 | **0.002** | 3.09 | 1.72 to 5.55 | **0.001** |
| **MMP9:** | **GG** | 1 (ref) |  |  |  |  |  |
| **rs17556** | **GT** | 0.84 | 0.27 to 2.63 | 0.758 | 1.10 | 0.30 to 3.96 | 0.890 |
|  | **TT** | 2.49 | 0.62 to 10.07 | 0.201 | 1.76 | 0.33 to 9.45 | 0.511 |
| **MMP9:** | **CC** | 1 (ref) |  |  |  |  |  |
| **rs2250889** | **CG** | 0.96 | 066 to 1.39 | 0.810 | 0.94 | 0.61 to 1.43 | 0.767 |
|  | **GG** | 1.29 | 0.62 to 2.68 | 0.490 | 1.99 | 0.86 to 4.62 | 0.111 |
| **MMP13:** | **AA** | 1 (ref) |  |  | 1 (ref) |  |  |
| **rs2252070** | **AG** | 1.15 | 0.75 to 1.77 | 0.521 | 1.29 | 0.77 to 2.16 | 0.334 |
|  | **GG** | 2.85 | 1.02 to 4.12 | **0.005** | 2.02 | 1.13 to 3.60 | **0.017** |
| OS, overall survival; MMP, matrix metalloproteases; HR, hazard ratio; ref, reference. | | | | | | | |
| # Adjusted for age, gender, histologic grade, gross type, tumor location, TNM stage and chemotherapy regimens. *p<0.05 was considered significant and were depicted in bold. HR and P values are obtained by multivariate Cox Proportional analysis. | | | | | | | |
|  |  |  |  |  |  |  |  |
